# Digital psychiatry and COVID-19: a potential recruitment opportunity

**DOI:** 10.1192/j.eurpsy.2021.132

**Published:** 2021-08-13

**Authors:** D. Rigby

**Affiliations:** The Meadows Inpatient Unit, Surrey and Borders Partnership NHS Trust, Epsom, United Kingdom

**Keywords:** digital, telepsychiatry, COVID-19, Recruitment

## Abstract

Psychiatry has long been battling with a recruitment crisis in the UK which is also reflected across much of Europe. Covid-19 has brought about widespread changes to our ways of working, as well as driving technological developments, which provides potential opportunities for the profession to draw people into the speciality. Covid-19 has brought interest in digital psychiatry from the peripheries to the mainstream. Mental health professionals are currently using sophisticated technologies such as Virtual Reality, Artificial Intelligence and Natural Language Processing in the diagnosis and treatment of mental health disorders. Highlighting the ways in which our profession is at the cutting edge of innovation to junior doctors offers a fruitful avenue to improve recruitment into the speciality. Many outpatient clinics have made the move to online service delivery during the pandemic to varying degrees. For many clinicians this has allowed more flexible and efficient ways of working. Psychiatry is better placed than most other medical disciplines to retain online patient contact in future clinical practice, post pandemic and may provide an attractive proposition for future psychiatrists. This talk will review some of the ways in which developments in digital psychiatry have been used to help generate interest for recruitment into the discipline as well as evaluating the benefits and challenges of the shift to telepsychiatry during Covid-19 and will offer some suggestions what the profession can learn from this to help future recruitment.

**Disclosure:**

No significant relationships.

